# SKIN-TO-SKIN CONTACT AND BREASTFEEDING AT CHILDBIRTH: WOMEN’S DESIRES, EXPECTATIONS, AND EXPERIENCES

**DOI:** 10.1590/1984-0462/2022/40/2020140

**Published:** 2021-05-26

**Authors:** Alice Parentes da Silva Santos, Zeni Carvalho Lamy, Maria Eduarda Koser, Clarice Maria Ribeiro de Paula Gomes, Beatriz Matos Costa, Laura Lamas Martins Gonçalves

**Affiliations:** a Universidade Federal do Maranhão, São Luís, MA, Brazil.

**Keywords:** Pregnancy, Humanizing delivery, Term birth, Postpartum period, Breastfeeding, Gestação, Parto humanizado, Nascimento a termo, Puerpério, Amamentação

## Abstract

**Objective:**

To analyze women’s desires, expectations and experiences regarding skin-to-skin contact and breastfeeding in the first hour of life of their newborns.

**Methods:**

Qualitative research carried out in a teaching hospital in the Northeast Region of Brazil. The patients were followed longitudinally during prenatal care, at birth and during the puerperium. The participants were pregnant women during normal risk prenatal care, aged over 18 years old. Structured and semi-structured interviews were carried out in the prenatal period, participant observation at the time of delivery and new interviews in the puerperium. Content analysis was applied in the thematic modality.

**Results:**

18 women between 21 and 38 years old were enrolled in the research. Women expressed the desire for skin-to-skin contact and breastfeeding as immediate practices right after delivery and birth. However, many women did not believe it was possible, and the performance of routine procedures was considered the main obstacle. These expectations that skin-to-skin contact and early breastfeeding would not be carried out were confirmed in the experiences immediately after birth.

**Conclusions:**

The expectations and experiences brought by these women suggest a flaw that starts in prenatal care and implies difficulties in implementing the studied practices. Thus, the empowerment and participation of women can become an important tool in the humanization of birth.

## RESUMO

**Objetivo:**

Analisar os desejos, as expectativas e as experiências de mulheres no que diz respeito ao contato pele a pele e à amamentação na primeira hora de vida.

**Métodos:**

Pesquisa qualitativa realizada em um hospital de ensino de uma capital da Região Nordeste. As mulheres foram acompanhadas longitudinalmente, durante o pré-natal, o parto e o puerpério. As participantes eram gestantes em pré-natal de risco habitual, com idade maior de 18 anos. Foram realizadas entrevistas estruturadas e semiestruturadas no pré-natal, observação participante no momento do parto e novas entrevistas no puerpério. Fez-se a análise de conteúdo na modalidade temática.

**Resultados:**

Participaram da pesquisa 18 mulheres, entre 21 e 38 anos. Elas expressaram o desejo do contato pele a pele e da amamentação como práticas imediatas após o parto e o nascimento, contudo muitas não acreditavam que fosse possível, sendo o principal entrave a realização de procedimentos de rotina. As expectativas de impossibilidade do contato pele a pele e amamentação precoce foram confirmadas no momento do parto.

**Conclusões:**

As expectativas e experiências trazidas pelas mulheres apontam para uma falha que se inicia no pré-natal e implica dificuldades na implementação das práticas estudadas. Desse modo, o fortalecimento da participação das mulheres pode se mostrar uma ferramenta importante na humanização do nascimento.

## INTRODUCTION

Skin-to-skin contact (SSC) and breastfeeding in the first hour of life (BFH) are practices that help reduce neonatal morbidity and mortality.^1^ ^,^ ^2^ They stand out as benefits for the newborn (NB) cardiopulmonary stabilization, reduction of risk of hypoglycemia, hypothermia^3^ and infection^1^, and improve the rates of continued breastfeeding.^1^ ^,^ ^4^ ^,^ ^5^ For the mother, there is a reduction in anxiety and bleeding after childbirth, among other factors.^1^

The importance of stimulating SSC in the FHL as immediate practice for newborns who are in adequate clinical conditions is emphasized,^6^ putting other care as secondary, as one seeks to ensure humanized care at childbirth and in the puerperium.^2^ ^,^ ^7^

The advantages of SSC and BFH are to be found in Ordinance No. 371, which instituted guidelines for the organization of comprehensive and humanized care for newborns,^6^ but their promotion is still a challenge in Brazil, especially in the northeast region.^8^ The network “Cegonha” (Rede Cegonha, in Portuguese), implemented in 2011, defined the good practices for delivery/childbirth in Brazil,^7^ but the effects of this strategy on women’s desires and expectations when it comes to SSC and BFH are still unknown, assuming the importance of prenatal care in this construction and, consequently, in their experience during childbirth and in the puerperium.

That being said, this paper aimed to learn and analyze women’s desires, expectations and experiences related to to SSC and BFH at the time of delivery/childbirth.

## METHOD

This was a qualitative research conducted between May and November 2016 in a public maternity hospital in northeastern Brazil, accredited as Hospital “Amigo da Criança”. Data collection took place in three moments: pregnancy (M1), delivery (M2) and puerperium (M3). Prenatal women of habitual risk, over 18 years of age, with gestational age (GA) from 29 weeks determined by ultrasound in the first trimester, were included, considering that, during this period of pregnancy, concrete thoughts related to childbirth and the baby begin.^9^ Women with an intrauterine diagnosis of malformation and with mental disorders were excluded.

Knowing the complexity of this study, its design foresaw the non-adherence of all women to the three moments, especially to the moment of delivery, without prejudice to the final result, taking into account that, in qualitative research, more important than the final number of participants is the scope of the situations contemplated.

Data were collected by means of structured and semi-structured interviews, participant observation and analysis of medical records. The structured interview and medical records provided sociodemographic characteristics (age, level of education, occupation, monthly income, marital status, ethnicity), obstetric data (planning of pregnancy, number of prenatal consultations, number of pregnancies, deliveries and abortions, and type of delivery), and neonatal details (gestational age, sex, weight and Apgar score).

The semi-structured interviews, recorded and transcribed, were conducted with the same women in M1 and M3. A script with guiding topics was used for each moment. The questions in M1 script were related to orientations received during prenatal care and to the wishes and expectations regarding the SSC and BFH. The questions in M3 script involved the experiences at the time of delivery: perception of the moment of birth, first contact with the baby, SSC and BFH. Whenever requested, the researchers provided guidance on SSC and BFH after the interviews.

Participant observation took place at M2 and aimed at a better understanding of the environment and situations reported in the interviews. The data were recorded in a field diary.

Initially, all women (31) who, at that time, were undergoing prenatal care at usual risk were approached. The research objectives were presented to them, and the invitation to the study was made. Three women did not accept to participate in the investigation for reasons not reported. The name, telephone number, and GA of the 28 women who accepted were registered. At 29 weeks of pregnancy, as previously agreed, the invitation by telephone was renewed. At this stage, ten women withdrew from participation, claiming an overload of their routine related to the proximity of delivery. Thus, 18 took part in the first interview (M1).

Although the observation in M2 was not planned to occur in all deliveries, considering the challenges inherent to the timely communication of the beginning of labor, the request was made for all women interviewed, and 17 gave the authorization. A new telephone contact was scheduled to the 36th week of pregnancy, for agreements related to the presence of the researcher during childbirth, which happened in four cases.

**Figure 1:**
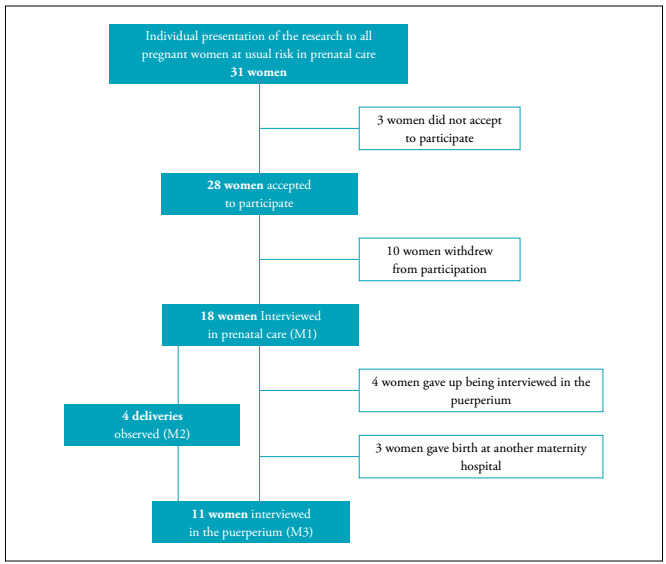
Flowchart of women’s participation in the study.

The second interview (M3), took place with 11 of the 18 interviewees in M1 (three women delivered in other maternities and four indicated unavailability to continue the interviews). The flowchart of participants is shown in Figure 1. This final number of interviews met the saturation criterion, a way of measuring the number of interviews in qualitative research.^10^

Content analysis was used in the thematic modality,^11^ which makes it possible to highlight the core meanings of communication^12^ according to the following methodological procedures: data organization, categorization and coding, inference and interpretation of results.^11^

The research was approved by the Research Ethics Committee (Certificate of Presentation of Ethical Appreciation - CAAE - 53596316.2.0000.5086). To guarantee anonymity of participants, their names were replaced after the names of Brazilian maternity hospitals.

## RESULTS

Interviews were conducted with 18 women in the prenatal period. Of these, four were observed at delivery and 11 were re-interviewed in the puerperium (Figure 1). The characterization of participants is shown in Chart 1.

**Chart 1:** Characterization of women interviewed, São Luís, 2016 *.

Participants	Age	Level of education	Marital status	Ethnicity	Pregnancy planning	Current pregnancy	Pre-natal appointments	Type of delivery	AG	1- and 5-min Apgar score
Sofia	28	High School	Married	Brown	No	1st	8	C-section	38w 4d	9/9
Esperança	26	Higher Education	Single	Black	No	1st	6	C-section	37w 3d	5/6
Leide	38	Higher Education	Single	Black	No	1st	7	C-section	41w	9/9
Nazaré	21	High School	Single	Black	No	1st	-	-	-	-
Risoleta	22	Incomplete Higher Education	Single	Yellow	No	1st	7	Natural	39w 5d	9/9
Regina	23	High School	Stable Union	Black	Yes	1st	8	Natural	38w 3d	8/8
Balbina	30	High School	Stable Union	Black	Yes	2nd	-	-	-	-
Conceição	36	High School	Married	Black	Yes	1st	6	Natural	40w 4d	4/8
Evangelina	27	High School	Stable Union	White	No	2nd	6	C-section	38w	9/9
Ana	32	High School	Single	Brown	No	3ª	7	Natural	38w 1d	9/9
Amparo	22	Elementary School	Stable Union	Black	Yes	2nd	8	C-section	40w	8/8
Bárbara	35	High School	Stable Union	Brown	Yes	3ª	9	C-section	39w 3d	8/9
Leila	24	Incomplete Higher Education	Stable Union	Black	No	1st	10	Natural	39w 5d	9/9
Carmela	19	High School	Stable Union	Brown	No	1st	9	Natural	39w 6d	9/9
Amélia	24	Nursing Technician	Married	White	No	2nd	8	C-section	40w 5d	9/9
Mariana	33	Incomplete Higher Education	Single	Brown	No	2nd	7	C-section	38w 6d	9/9
Darcy	33	High School	Stable Union	Brown	No	4ª	-	-	-	-
Catarina	28	High School	Married	Brown	No	4ª	8	C-section	39w 3d	9/9

*Missing information is from women whose deliveries took place at a maternity hospital other than where the research was conducted; GA: gestational age; w: weeks; d: days

In the analysis of their speeches, two categories were identified: “I hope the baby comes soon into my arms…, but I don’t know if it is possible”, which includes women’s wishes and expectations about SSC and BFH; and “It’s very fast, we can’t even take a close look at the baby”, which includes experiences related to the studied practices.

“I hope the baby comes soon into my arms…, but I don’t know if it is possible”

Of the 18 pregnant women, 13 reported wishing to have their child in their arms and to breastfeed them immediately after birth. Despite this desire, they did not mention the term SSC, and their expectations were often that the NB would undergo routine procedures before the first contact and breastfeeding:

“I can already picutre her on top of me. Having her in my arms and being able to breastfeed her, you know? This is what I want and what I desire. [...] I hope it will be soon after she is cleaned, and everything. May they give her to me soon” [Amélia, M1].

“As soon as my son is born, I want to be the first person to hold him. Because we’ve been together for so long, I don’t think it’s fair for someone else to take him first. Although the nurse usually takes the babies first, I want to hold him, I want to know him, I want to touch him, hug him, kiss him, I want everything” [Leila, M1].

“I want them to show the baby to me right after he is out, then they can take him to all the procedures [...], clearing obstructed airways, cleaning, weighing, identification” [Sofia, M1].

If women expressed a desire to have their children in their arms shortly after birth and there is evidence of the benefits of SSC, why don’t they express that desire when they talk about what they expect to happen at the time of birth (expectations)?

This gap between expectation and what is recommended as good practice indicates lack of communication and health education in prenatal care. There is still the naturalization of the performance of procedures before SSC and BFH by women. Some reported the expectation that they would be with their children only after they are cleaned: “One thing I asked my mother: do not put him on top of me. Not dirty.” [Esperança, M1].

Regarding the expectations about what would happen immediately after birth, we highlight:

“That is what I saw and read in some places. I researched, I saw [...] that they will clean and weigh the baby, put the identification, and then they will bathe him. Only then will they bring him to the mother, so that I can feed him, and that’s it” [Risoleta, M1].

“In my previous pregnancies, it was like this: as soon as we went to the room, the nurse came to put her to breastfeed. [...] I think that if it is different, [...] I want everything to go well, I want her to go to the room with me, so that I can breastfeed her soon, when I get there” [Catarina, M1].

“Some friends told me that they don’t [...] give us the baby right away. First, they do [...] the procedures, clean the baby, then we receive them” [Leide, M1].

We could see that many of the expectations are built based on media sources and previous experiences. The participants did not mention prenatal care as a source of information about SSC and BFH.

“It’s very fast, we can’t even take a close look at the baby”

When analyzing the experience in the light of desires and expectations (Chart 2), it was evident that for many women the experience of SSC and BFH was different from what they had narrated in the first interview, considering that most NBs did not receive SSC and, in almost all situations, were only shown to the mothers and then taken for routine procedures, preventing prolonged contact, as reported:

“I wanted her to come closer, give her a kiss. If I didn’t have the devices in my hands, for sure I would have held her, you know? So I touched her head. [...] They had to take her to clean her” [Leide, M3].

“They come close to show you the baby... I think this is to give the mother some kind of relief. They show the baby, they examine everything. Then they told me they had to go wrap him in cloth, examine, weigh, measure, clean” [Catarina, M3].

**Chart 2:** Matching of wishes, expectations and experiences of the 11 interviewees in prenatal care (M1) and puerperium (M3).

Participants	Wishes (M1)	Expectations (M1)	Experience (M3)
Sofia	Seeing the child first and only then they be taken for cleaning, weighing and identification	The child being cleaned, bathed and dressed before first contact and breastfeeding	Looked at the child quickly in the delivery room; contact and breastfeeding between one and two hours after delivery, in the recovery room
Esperança	The child being cleaned, bathed and dressed before first contact and breastfeeding	The child being cleaned, bathed and dressed before first contact and breastfeeding	Breastfed in the recovery room after oxygen therapy
Leide	Having the child in their arms	The child being cleaned before first contact and breastfeeding	Looked at the child quickly after delivery; contact and breastfeeding in the recovery room, after cleaning of the newborn
Regina	Having the child in their arms	The child being cleaned before first contact and breastfeeding	Quick SSC and subsequent separation due to the need for oxygen therapy. Breastfed after reunion
Ana	Having the child in their arms and being around during their cleaning.	Having the child in their arms and being around during their cleaning.	Quick approach with newborn wrapped in cloth immediately after delivery and subsequent separation for cleaning of the child. Breastfed after reunion
Amparo	Having the child in their arms	The child being cleaned before first contact and breastfeeding	Looked quickly at the child after delivery, and the newborn was taken to the NICU. Breastfed at the NICU, one day after birth
Bárbara	Having the child in their arms and being around during their cleaning.	Having the child wrapped in cloth and breastfeed	Reported not remembering what happened to her child after giving birth. Pointed out forgetfulness due to the effect of the anesthesia. Breastfed in the recovery room.
Leila	Having the child in their arms	Having the child examined by the medical staff.	Quick approach with the child wrapped in cloth and separation for evaluation and cleaning
Amélia	Having the child in their arms and being around during their cleaning.	The child being cleaned before first contact and breastfeeding	Looked at the child quickly after delivery; breastfed in the recovery room after cleaning
Mariana	Having a cesarean section and breastfeed the child after recovery from anesthesia	Having a cesarean section and breastfeed the child after recovery from anesthesia	Breastfed the baby wrapped in cloth in the recovery room, after examinations and evaluation
Catarina	Having the child examined, evaluated and cleaned before first contact and breastfeeding	Having the child examined, evaluated and cleaned before first contact and breastfeeding	Looked at the child quickly after delivery; breastfed the child wrapped in cloth in the recovery room, after weighing, cleaning, and measuring height

SSC: skin to skin contact; NB: newborn; NICU: neonatal intensive care unit.

Of the eight women who delivered via c-section, none experienced SSC immediately after delivery, according to them, because of the said need to perform routine procedures, such as cleaning, weighing and measuring the newborn. The three women who had natural delivery received their baby in their arms at the time of birth. In one of the situations, the newborn was taken because he needed oxygen. In the other two cases, the contact was not skin-to-skin; there was a sheet between the mother and the newborn. Still, women who had a natural delivery experienced greater contact with their children and reported this experience as positive.

The first physical contact between mother and baby after c-section delivery occurred mostly in the recovery room, between one and two hours after birth, when they first breastfed:

“I don’t think it took long. About one to two hours. [...] So, as soon as they put her there, she took my breast. [...] She was wrapped in a cloth” [Sofia, M3].

The participative observations allowed to learn this reality: the NB was delivered to the mother in the recovery room, within 51 to 57 minutes after birth.

In these observations, we highlight the role of the nursing team, which provided initial guidance on breastfeeding; however, they did not point out the SSC as an important practice. BFH was a more stimulated practice when compared to SSC. According to the interviewees, the duration of SSC varied from 1 to 15 minutes, being interrupted due to the NB’s need for episiorrhaphy or respiratory aid.

The first breastfeeding was marked, in cases of cesarean section, by difficulties related to the mother’s limited mobility, but there was encouragement by professionals:

“I couldn’t touch her, because I was recovering from anesthesia. I couldn’t move around much. [...] Then, they put her close to me and said: ‘The baby is going to breasfeed. She was lying here beside me, and they pulled my breast for her to breastfeed, it wasn’t me who did it.” [Barbara, M3].

The participative observations showed us that, even in the face of such difficulties and discomforts, women showed themselves available to breastfeed their newborns.

## DISCUSSION

The desires for immediate contact with the child and breastfeeding after birth expressed by pregnant women are in accordance with the practices recommended in the literature,^1^ ^,^ ^2^ ^,^ ^3^ ^,^ ^6^ ^,^ ^13^ ^,^ ^14^ but the fact that these desires were not expressed as expectations can demonstrate that the performance of routine procedures at the time of birth is still very present in the imaginary of women and, also, that the guidelines received in the prenatal consultations did not allow the desire to become an expectation.

Although it is known that, when the NB is healthy at birth, all routine procedures should be postponed and some even abolished, the idea that the baby needs to be “cleaned” at birth, neglecting the protective effect of the vernix, it is also present among professionals.^15^

It is important that measures to prioritize SSC and BFH are adopted, postponing or eliminating interventions at birth.^13^ In the present study, some procedures that represent an inadequate care model in the light of current scientific knowledge were still reported as wishes and/or expectations by interviewed women. Thus, the importance, of guidelines on proven beneficial practices in prenatal care should be reinforced.

Regarding the sources of information about practices related to childbirth, the following stand out: the media,^2^ previous experience and the report of women from their social support network.^16^ In the present study, the media and previous experiences were the basis for the production of expectations, according to reports. In this sense, the importance of prenatal care is reinforced as an opportune time and place for women to access content of adequate quality and good understanding, as a way to contribute to better expectations and positions that leads them to pressure for the improvement of childbirth assistance.^17^

Why do women who do prenatal care reach the end of pregnancy with so much misinformation about SSC and BFH? Probably, the information about these practices and their advantages either is not given or is given in a way that do not produce any meaning for pregnant women.^18^ We highlight that the publication “Caderneta da Gestante” (Pregnant Woman’s Notebook),^19^ by the Ministry of Health, should be made available during prenatal care, as it brings this information. We also highlight that the participants of this study had prenatal care and had their births in a maternity hospital accredited by the Baby Friendly Hospital Initiative (IHAC), a strategy that encourages SSC and BFH.^2^

It is up to the assistance team to explain the hospital’s routines and the benefits of SSC and BFH. This way, women are provided with better knowledge about the moment of delivery and can claim the fulfillment of their wishes, which, when converging with good practices, must be recognized and legitimized as a right.

Studies show that the guidelines received during pregnancy on SSC are scarce, which puts the spotlight on the social and family support network, groups of pregnant women and the obstetric center as important spaces for the sharing of information about this practice.^2^ ^,^ ^4^

Another important aspect related to the pregnancy in this study refers to the absence of reports of a birth plan, an important strategy recommended by the World Health Organization (WHO), since 1996, which encourages the search for information, and thus helps to build expectations and desires associated with the experience of motherhood as a protagonism exercise.^20^ Guidance on birth plan is also mentioned in “Caderneta da Gestante”.^19^

Studies have reported that the approximation between mother and child has been delayed by cesarean deliveries^8^ ^,^ ^21^ and labor analgesia.^22^ This may have had a negative impact on the practice of SSC in this investigation. The survey “Nascer” in Brazil, which used a representative sample of 23,940 women from across the country, also indicated that SSC was less frequent after c-section deliveries than after natural deliveries.^23^

The routine procedures reported as expectations of women and experienced in their experiences are inconsistent with the recommendations of good practices based on evidence,^7^ ^,^ ^24^ which highlight the importance of prioritizing SSC and BFH before any other interventions.^13^

Apgar scores above 8 are shown to be a protective factor for SSC and BFH.^22^ In addition, clinical stability at birth supports the recommendation for the practice of SSC and BFH.^1^ ^,^ ^6^ However, in this study, in some cases, the practices were little effective even in newborns with Apgar greater than 8, suggesting that the actions continue to be postponed and placed as secondary to routine procedures, reinforcing the importance of their better implementation.

The finding that BFH was more stimulated than SSC is similar to the results reported in the survey “Nascer”,^23^ which found, in capitals and maternity hospitals of the northeast region linked to IHAC, higher rates of BFH than SSC. This may indicate that practices such as weighing and assessing the newborn are being carried out before SSC and only after that the baby returns to the mother for breastfeeding.

With regard to the duration of the SSC, our results are similar to those of another research^8^, in which few women experienced SSC and, of these, an even smaller proportion remained in SSC for more than 30 minutes or until the moment of first breastfeeding.

Natural delivery brought mothers and the NB closer to experiencing immediate SSC, however the use of cloths between them and the reduced time of the practice are elements that deserve attention.

Thus, this research concluded that most women’s desires were different from their own expectations and experiences. Women still expect a model of care during childbirth that disregards their autonomy and their role, and this is reinforced by experiences.

Despite national actions that encourage good practices in childbirth, the results of this research showed that investments are still necessary for its full implementation. Prenatal care appointments were not a space for orientations that could contribute to the building of expectations that involve recommended and useful practices. Women informed about their rights strengthen the movement in defense of the humanization of childbirth and are an additional factor to pressure services to implement humanized practices in the Unified Health System.

The main limitation of this study was the difficulty in keeping women in the research, from the beginning of pregnancy to the puerperium. Despite the initial acceptance, due to issues related to the moment of life, some women had difficulties in scheduling the interviews throughout the follow-up. Another limitation was the difficulty in observing delivery (M2). Even so, of the 18 women interviewed in the prenatal period, four birth were watched and 11 women were interviewed in the puerperium, taking into account the perspective of qualitative research.

It is hoped that these findings will allow reflections and changes in prenatal and childbirth care practices, enabling women with desires, expectations and experiences related to SSC and BFH based on good practice guidelines.
